# The Impact of Incorporating Multiple Best Practices on Live Outcomes for a Municipal Animal Shelter in Memphis, TN

**DOI:** 10.3389/fvets.2022.786866

**Published:** 2022-06-24

**Authors:** Rachael E. Kreisler, Alexis A. Pugh, Katie Pemberton, Sara Pizano

**Affiliations:** ^1^Department of Primary Care, Midwestern University College of Veterinary Medicine, Glendale, AZ, United States; ^2^Memphis Animal Services, Memphis, TN, United States; ^3^Team Shelter USA, Coral Springs, FL, United States

**Keywords:** managed intake, safety net program, live outcome, field services, Shelter-Neuter-Return, community cat, return to field, live release rate (LRR)

## Abstract

Modern animal shelters are encouraged to adopt “best practices” intended to promote life-saving for the animals that enter their systems. While these best practices have been defined and widely promoted within the profession, few studies have tracked how making the recommended changes affects live release rates (LRR) and other shelter metrics. In 2017, the municipal animal shelter in Memphis, TN (Memphis Animal Services) implemented five new strategies and analyzed their resultant life-saving data. The interventions included managed strategic shelter intake, pet owner safety net, community cat return to field, transition of field services from punitive to assistive, and streamlined adoption and transfer protocols. The median LRR for cats prior to 2017 was 35% (IQR 22, 36). After the intervention, the LRR increased to a median of 92% (IQR 92, 94). The correlation between intake and euthanasia for cats prior to the intervention was significant (*P* < 0.001) and very strong (*r* = 0.982), while after there was no relationship (−0.165) and it was not significant (*P* = 0.791). The median LRR for dogs prior to 2017 was 25% (IQR 19, 48). After the intervention, the LRR increased to a median of 87% (IQR 86, 88). The correlation between intake and euthanasia for dogs prior to the intervention was significant (*P* < 0.001) and very strong (*r* = 0.991), while after there was a moderate relationship (−0.643) that was not significant (*P* = 0.242). The median LRR for kittens prior to 2017 was 34% (IQR 23, 38), which increased (*P* = 0.001) to 92% (IQR 91, 92) after intervention. The percent of kittens entering the shelter with an outcome of euthanasia decreased (*P* < 0.001), from a median of 59% (IQR 54, 73) to a median of 3% (IQR 1, 3). The median return to owner (RTO) rate for dogs increased (*P* = 0.007) from 10% (IQR 9, 11) to 13% (IQR 13, 13). Implementation of these best practices accelerated Memphis Animal Services' progress toward a live release rate of at least 90%, particularly for cats, dramatically decreased kitten euthanasia, increased the RTO rate for dogs and severed the historical correlation between euthanasia and intake.

## Introduction

Animal shelters in the United States were historically created with the primary objective of protecting human health, particularly from rabies. Shelter facilities and protocols, particularly those operated by municipalities, were designed to accommodate stray animals (those that have strayed from home and become lost or that have been abandoned) for a brief holding period followed by euthanasia for unclaimed animals (“catch and kill”). Historically, many municipal shelters operated as an “open admission” system, meaning all owner surrendered and stray pets were admitted to the shelter with no attempt at mitigation. If the shelter was full or over capacity, euthanasia was used as a tool for population management to make space for new incoming animals (1).

In the 1970s, an estimated 20 million animals entered shelters and 13.5 million were euthanized (2). Since then, societal views regarding animals have evolved and canine rabies variant has been eradicated from the United States due to vaccination and animal control measures (3, 4). Communities subsequently desire live outcomes for shelter animals and modern animal shelters are able to focus on other goals such as life saving. However, finding the resources to provide live outcomes for most animals admitted to the shelter can be challenging for shelters evolving from a catch and kill model to one that supports the goals of a modern animal shelter. These goals include providing live outcomes for pets who do not have owners or must be rehomed (5) and reuniting lost pets with their owners (6). The rate of live outcomes, also known as live release rate, is often used as a benchmark, with a rate of 90% or greater generally targeted, as it suggests that animals are not euthanized for population management (7). It is also important that modern animal shelters provide a high standard of animal welfare for animals within the shelter's care and engage constructively and collaboratively with their community (8).

Returning lost pets to their owner is another primary goal of a modern shelter. Return to owner (RTO) rates are highly variable between communities, although it is very consistent that the cat RTO rate is approximately an order of magnitude less than dogs within a given community (7). This finding may be due to the differing ways in which lost cats and dogs are reunited with their owners, with the greatest proportion of dogs being reunited via a shelter, and the greatest proportion of cats finding their way home on their own (9, 10). The nationwide average RTO rate is estimated to be 19% for dogs (11). RTO rates are calculated by dividing the number of pets reclaimed by their owner by the number of stray pets entering the shelter (12).

Cats are generally not well-served by traditional shelter practices. This problem is due to a relatively large number of cats entering the shelter, differences in the way that cats as compared to dogs are acquired as pets, and the fact that community cats can sustain themselves. Community cats are those that are free-roaming (not confined in a house or other type of enclosure) and may be socialized or unsocialized (untamed or feral). Although cats are slightly less than half of shelter intake, it is estimated that they are euthanized for population management at a ratio of more than 2:1 as compared to dogs (7). Historically, intake and euthanasia were tightly coupled, with a correlation of 0.964 found consistently across multiple states with variable levels of per-capita intake during the period of 2003 to 2007 (13). While pet acquisition statistics vary between sources, cats are consistently acquired directly more frequently as strays as compared to dogs (2). Community cats, whether social or not, are commonly able to maintain themselves either by scavenging or *via* support from human caretakers resulting in a large population of cats perceived as stray, particularly kittens. Even for cats that are maintained by caretakers or loosely owned, many caretakers or semi-owners would be unlikely to look for their cat in a shelter if they were to go missing for a few days (14). This fact has consistently resulted in a return to owner rate for cats entering a shelter categorized as stray of <3.5% annually as compared to 22% of dogs (7).

Several innovative strategies aimed at the efficient use of shelter resources to meet the modern goals for animal shelters and implemented at various shelters have been promoted as “best practices” within the profession (8). Strategies such as managed strategic intake, pet owner safety net programs, community cat return to field, transition of field services from punitive to supportive, and streamlined adoption and transfer show great promise but have not been fully analyzed within the scientific literature.

Managed strategic intake regulates or schedules non-urgent intake to the shelter so that all viable alternatives to shelter intake are exhausted before an animal enters the shelter. Shelter space is a crucial resource and by using it only for pets with no other options, more pets and people can be helped. When shelter space is reserved for those pets with no other alternatives and there are fewer pets in the shelter to care for, the staff is better able to serve and provide for the pets that do enter. Managed intake helps shelters to plan for appropriate staffing and ensure that there is the capacity to serve the animals that enter the shelter. It also involves researching and providing resources outside of the shelter to pet owners who are experiencing challenges keeping their pet in their home (8, 15, 16).

Safety net programs are designed to assist pet owners in need or help pet owners rehome their pets directly in lieu of shelter intake (17). The shelter provides direct or referral services to help pet owners avoid the need to surrender their pet or to support them in adopting their pet to another home without a stay in the shelter (8).

Return to field (RTF) or Shelter-Neuter-Return programs provide a live outcome for healthy community cats categorized as stray with good body condition. These cats are sterilized, vaccinated, and returned to the location where they were found (8). RTF is similar to Trap-Neuter-Return (TNR), with the main difference being that the cats in RTF programs have undergone intake to the shelter as a stray, as compared to TNR where cats are trapped for the specific purpose of sterilization and the procedures provided as a clinical service. However, RTF programs have been shown to increase the live release rate for cats while decreasing the amount of time that they spend in the shelter (18, 19).

Traditionally animal control services have operated with a punitive enforcement-minded approach and primary duties have included issuing citations for animal-related infractions, transporting non-aggressive, healthy, free-roaming animals to the shelter for intake, and seizing animals. However, this approach is not conducive to the goals of a modern shelter, particularly reuniting lost pets with their owner and engaging collaboratively with the community. Emerging data in Dallas (12), El Paso and Austin, Texas, have demonstrated that animals picked up by an animal control officer (ACO) are typically found very close to their home, suggesting that animals may be more likely to be reunited with their family if efforts are made to locate the owner prior to transporting them to the shelter. Transforming field services into an assistive rather than punitive role also encourages constructive interaction with the community, helping to build trust and a collaborative relationship with the shelter.

Two common live outcomes for many shelters are adoption and transfer to another shelter or rescue organization with different resources or demand for animals. However, there has traditionally often been barriers to adoption such as long adoption applications with very specific requirements for housing (20). Transfer fees charged to organizations accepting transfers have been levied in an attempt to recoup the costs of impounding an animal or loss of potential adoption income, resulting in barriers to the transfer out of animals.

### Background

In January 2016, the new mayor elected in the city of Memphis, Tennessee was given a clear mandate by the community to commit Memphis Animal Services (MAS), the local government shelter operated by the city of Memphis, to a focus on life-saving (21). Between 2008 and 2015, MAS had a historical live release rate ranging from 9 to 65% (22). Several categories of animals, such as community cats and neonates, were euthanized on intake despite most being healthy on presentation.

Those historical policies were coupled with other barriers to live outcome, including the requirement for an ACO to do a home visit and fence check for the adoption of a pit bull-type dog as well as background checks for those interested adopters. Although the shelter worked with non-profit rescue groups interested in transferring and saving animals, the $50 charge per pet posed a financial barrier to those groups.

At the time, MAS was under the Parks and Neighborhoods department with several layers of decision-makers between the shelter administrator and the mayor. The new mayoral team decided to create an independent department and reclassify the shelter administrator position to a director position answering directly to the Chief Operating Officer under the mayor.

Soon after the mayoral election, a new director was hired and Target Zero, a charitable initiative offering pro bono shelter and community assessments nationally between 2013 and 2017, was invited to complete a shelter assessment. The Target Zero team (which included one of the authors, SP) provided a report to benchmark national best practices compared to current MAS protocols. They created a plan for a progressive animal welfare system that would increase lifesaving, increase animal welfare, and fulfill the goals of a modern animal shelter. This plan included the implementation of managed strategic shelter intake and a safety net program with the goal of only admitting animals that require and are benefited by intake to the shelter. The new administration also embraced simplifying the adoption process and eliminating unnecessary adoption barriers like home visits and fence checks.

The purpose of this study was to document the impact of these key best practices on lifesaving, animal welfare, and modern sheltering goals.

## Methods

### Description of Interventions

#### Managed Strategic Shelter Intake

The Target Zero consultation included an examination of statutes and contracts that determined that the shelter was not legally required or mandated to accept owner surrendered pets. The managed strategic shelter intake program began in 2017, with the first step being the requirement of an appointment for non-emergent owner surrenders. Emergency cases could still be admitted without delay if necessary.

#### Pet Owner Safety Net

Initially, there was no budget for a formal Safety Net assistance program to directly provide resources to the public, so leadership focused on linking pet owners to information and other resources available outside the shelter. A Skip the Shelter brochure was created that listed rescue partners, pet-friendly housing options, low-cost spay/neuter programs, and information about Care Credit for those needing veterinary care at a private clinic (Supplementary Addendum 1). Prior to 2017, neonates were typically euthanized upon intake because there were no resources to care for them in the shelter. As part of the Safety Net Program, MAS educated the community about neonatal kittens. Educational information was provided on the website to direct finders of neonatal kittens to leave them in place or to return nursing kittens where they found them when not at risk. Finders of kittens requiring a foster home were provided educational materials regarding caring for underage kittens and supplies (Supplementary Addendum 2).

In 2020 the shelter expanded the Safety Net program to include a Pet Resource Center (PRC) to use shelter-provided resources to assist pet owners in need as well as address other types of shelter intake. The PRC became an integral part of the MAS budget, with coverage for the two full-time Pet Resource Specialist positions as well as subsidies to help pet owners and finders. The PRC is additionally supported by grants and donations. Leadership determined the subsidy amount of $300 that PRC resources specialists may approve to prevent a surrender based on the estimated cost of $309 to admit a pet to the shelter. This amount was calculated by dividing the average annual intake into the overall operating expenses less field operations. Financial assistance may cover veterinary care, a temporary stay at a boarding facility, pet deposits for housing, fixing fences, behavior training, pet food, free spay/neuter or whatever intervention may help the owner keep their pet. Shelterluv^1^ software is used to track the work of the specialists using their free field and community services platform.

#### Community Cat Return to Field

Prior to 2017, MAS euthanized most community cats on intake like many traditional municipal shelters at the time. It was calculated that sterilizing and returning healthy stray cats the following day to the location they were found cost < $150 as compared to the $309 per pet calculated for a typical intake. Implementing an effective community cat program began with training the staff and providing the tools they needed to explain the program to the public. One of those tools was a brochure that included Frequently Asked Questions and information was also added to the website (Supplementary Addendum 2). Staff were trained to have a conversation with constituents calling about or bringing a community cat to the shelter for the purpose of intake to explain how sterilization mitigated unwanted behaviors associated with mating, and to determine whether other resources were required to address concerns. Stray cats and kittens were evaluated on intake for the best pathway for the cat and shelter capacity. Kittens and socialized adult cats were put on an adoption track if shelter capacity allowed, if the kitten was too young for sterilization surgery, or if the cat could not be returned to its originating location. Constituents were asked if they were willing to return their community cat the day after the surgery. If the finder was unwilling or unavailable to do so ACOs returned the community cats.

#### Field Services Transition

Redefining the role of field services was an important step to meeting the goals of a modern shelter. The only pathway for assistance at the time was to admit an animal to the shelter. With the traditional approach, Animal Control Officers apprehended a dog at large and transported them to the shelter for admission and a stray hold period. Given MAS' catchment, this protocol meant that dogs might be transported up to an hour away from their home to the shelter. It is likely that many owners would not know about the shelter or that their missing dog would have been taken there. If an owner came forward, they were subject to citations, fines, or boarding fees prior to reclaiming. However, dogs at large who are not a public safety threat are a prime example of an animal that may not be best served by intake to a shelter.

Since 2019, MAS protocols have specified that ACOs must make all reasonable efforts to reunite dogs in the field and are instructed to spend time in the neighborhood, speak to neighbors, knock on doors, and speak to children playing outside to find the owner. If an owner is located the dog is returned without undergoing intake to the shelter. An informational door hanger is left on the house or houses where the ACO suspects the dog lives if the ACO must transport the dog to the shelter.

In 2020, the protocol for field services was revised to route field service calls through a specialist with the PRC before an ACO responds in-person to a dog at large call. The specialist discusses the possibility of the finder fostering the dog (Found Foster Program) and partnering in the efforts to locate the owner by checking for identification, placing flyers in the neighborhood, walking the dog in the area where they were found, and speaking to neighbors.

#### Streamlined Adoption and Transfer

Adoption and rescue transfer practices were streamlined. The requirement for a background check and an ACO home visit and fence check for the adoption of a pit bull-type dog was removed. The $50 charge per pet transferred to rescue was eliminated and staff created a more welcoming environment for the public and rescue groups.

### Statistical Methods

Descriptive statistics were used to summarize the shelter data, with the mean and standard deviation (SD) used for normally distributed data and the median and interquartile range (IQR), reported as (Q1, Q3) to describe the skew of the data, used for non-normally distributed data. Linear regression was used to determine the rate of change over time. T-tests were used to compare normally distributed data and Wilcoxon rank-sum tests non-normally distributed data before and after the intervention in 2017. Interrupted time series were used to compare trends before and after the intervention. The final disposition based live release rate was calculated as (live outcomes/all outcomes × 100) (23).

## Results

### Live Outcomes for Animals Entering the Shelter

**Figure 1:**
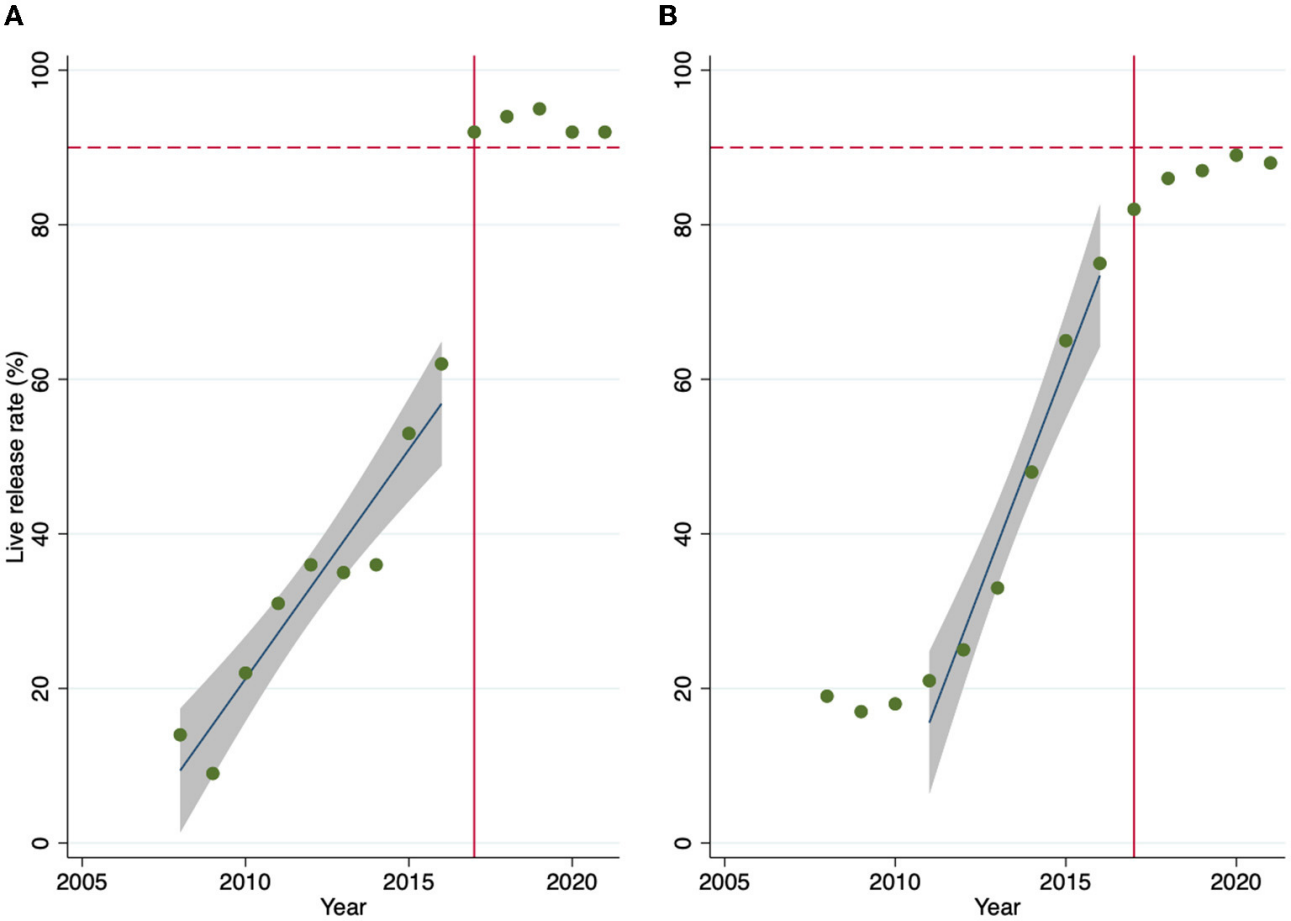
Live release rate for cats **(A)** and dogs **(B)** by year overlaid by best fit line and 95% confidence interval for years 2008 to 2016 for cats and 2011 to 2016 (the linear portion of the sigmoid curve) for dogs. Dotted red line at the 90% target live release rate and solid red line at the intervention year.

The median live release rate for cats prior to 2017 was 35% (IQR 22, 36). The live release rate was found to increase by 6% each year from 2008 to 2017 (*P* < 0.001), reaching a maximum of 62% in 2016—(Figure 1A). After the intervention in 2017, the live release rate increased to a median of 92% (IQR 92, 94)%. The correlation between intake and euthanasia for cats prior to the intervention was significant (*P* < 0.001) and very strong (*r* = 0.982), while after there was no relationship (−0.165) and it was not significant (*P* = 0.791).

The median live release rate for dogs prior to 2017 was 25% (IQR 19, 48). The overall increase in live release rate followed a sigmoid pattern (Figure 1B), with the live release rate relatively flat from 2008 to 2010, then rapidly increasing from 2011 to 2016, before leveling out just below 90% for 2017 through 2021. Linear regression of the linear portion of the sigmoid curve from 2011 to 2017 found an increase of 11% per year (*P* < 0.001). After the intervention, the live release rate increased to a median of 87% (IQR 86, 88). The correlation between intake and euthanasia for dogs prior to the intervention was significant (*P* < 0.001) and very strong (*r* = 0.991), while after there was a moderate relationship (−0.643) that was not significant (*P* = 0.242).

**Figure 2:**
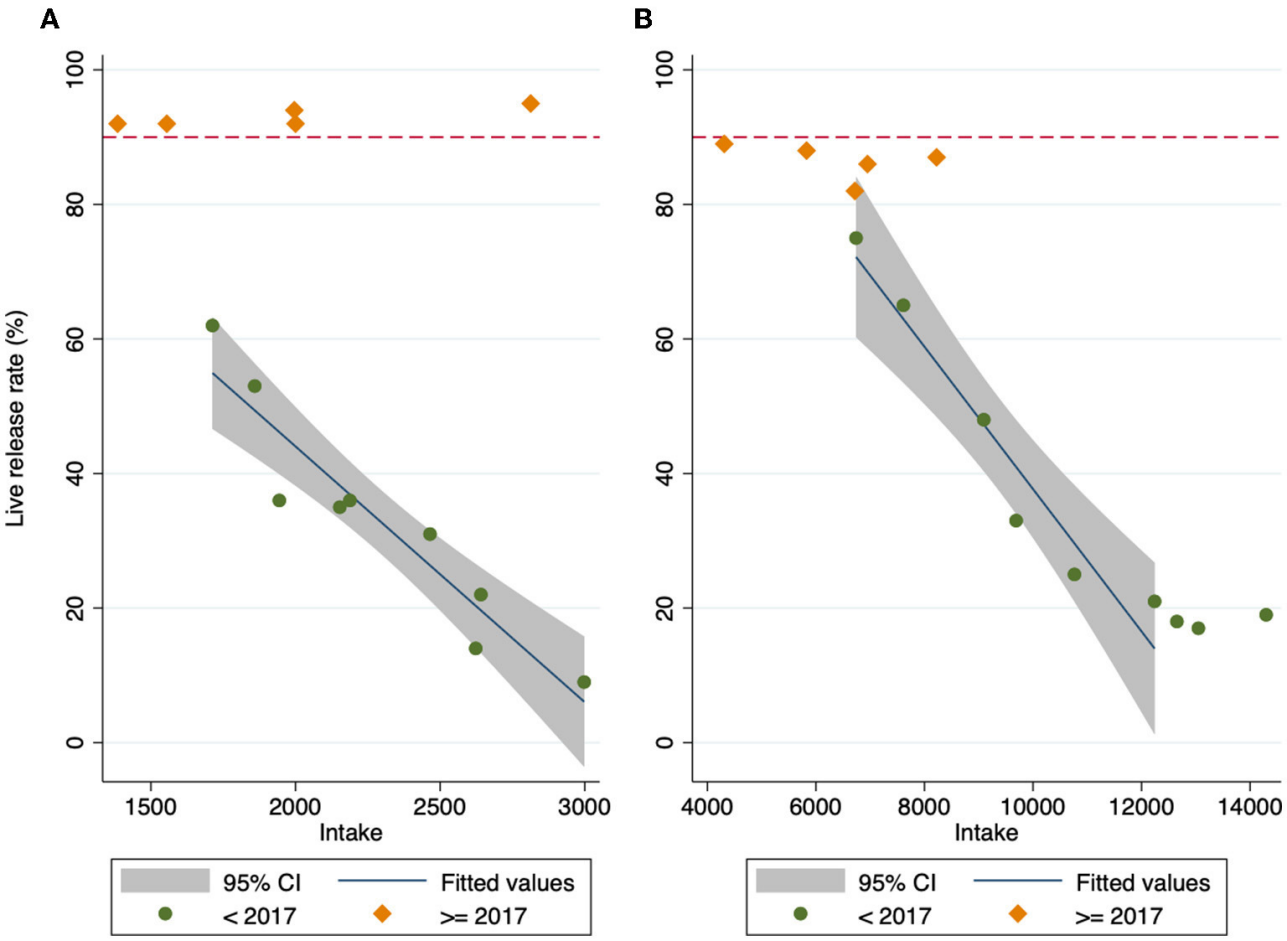
Live release rate for cats **(A)** and dogs **(B)** by the number entering the shelter. Intake prior to the intervention year (2017) in green, and intake from 2017 onward in orange. Dotted line at 90%. The best fit line for cats shows a linear relationship between intake and live release rate prior to 2017, while there is no relationship between intake and live release rate after. The best fit line for dogs showed a curvilinear relationship between intake and live release rate prior to the intervention year, while there is no relationship between intake and live release rate after the intervention year.

After the intervention the LRR was no longer correlated to intake, particularly for cats (Figure 2). Prior to 2017, intake and live release rate were tightly correlated. For cats, there was a linear relationship, with live release rate increasing by 4% for each fewer 100 cats entering the shelter (*P* < 0.001). For dogs, there was a sigmoid relationship, with the live release rate consistently low for intake >12,000, and an increase of 1% for each fewer 100 dogs entering the shelter (*P* = 0.001) in the linear portion of the sigmoid curve. After the intervention, there was no significant relationship between intake and live release rate for cats (*P* = 0.565) or dogs (*P* = 0.460).

#### Managed Strategic Intake

Cat intake prior to 2017 was a median of 2,188 (IQR 1,944, 2,623), with a decrease (*P* = 0.001) of 138 cats per year (Supplementary Addendum 3a). There was no significant change in intake after the intervention (*P* = 0.868).

Dog intake prior to 2017 was a median of 10,764 (IQR 9,093, 12,651), with a decrease (*P* < 0.001) of 937 dogs per year (Supplementary Addendum 3c). There was no significant change in intake after the intervention (*P* = 0.394).

### Pet Owner Safety Net

The composition of cat intake type prior to 2017 was a mean of 46% (SD 5) stray and 50% (SD 5) owner surrender, with the 4% remaining (enforcement for cruelty confiscation, bite or rabies quarantine, or born in care) classified as “other” (Supplementary Addendum 3b). After the intervention, the stray intake was a mean of 49% (SD 3), owner surrender was 29% (SD 5), and other (enforcement for cruelty confiscation, bite or rabies quarantine, or born in care) 22% (SD 3). The percent of owner surrenders decreased after the intervention [*t*_(12)_ = 8.1, *P* < 0.001], but not stray (P = 0.806) or other (*P* = 0.063).

The composition of dog intake type prior to 2017 was a mean of 63% (SD 4) stray and 28% (SD 3) owner surrender, with the remaining 8% (enforcement for cruelty confiscation, bite or rabies quarantine, or born in care) classified as “other” (Supplementary Addendum 3d). After the intervention, the stray intake was a mean of 69% (SD 3), owner surrender was 17% (SD 2), and other 14% (SD 3). The percent of owner surrender decreased after the intervention *t*_(12)_ = 7.3, *P* < 0.001, while stray and other intake increased, *t*_(12)_ = −2.6, *P* = 0.024, and *t*_(12)_ = −2.5, *P* = 0.029, respectively.

In 2021, the only year for which a full year of data from the fully operational PRC are available, the PRC handled 4,394 calls. Of these calls, 1,419 (32%) were for rehoming support, 1,223 (28%) were for assistance with pet food or supplies, 860 (20%) for assistance with medical care, 38 (1%) for assistance with behavior, and 854 (19%) other pet retention.

### Community Cat Return to Field

**Figure 3:**
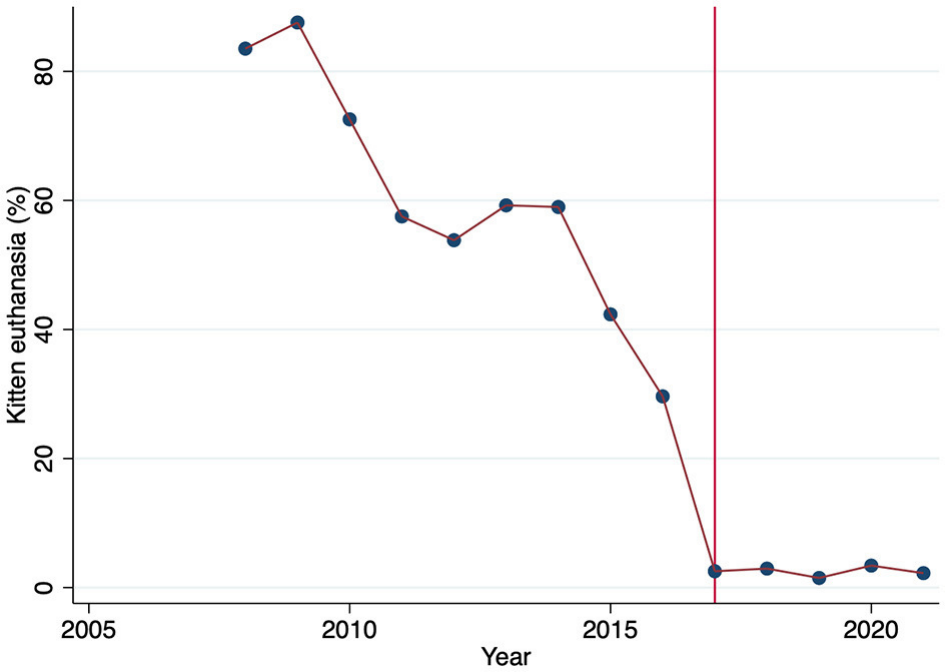
Percent of kittens entering the shelter <5 months of age euthanized by year. Solid line at intervention year.

The number of cats returned to field per year after the intervention ranged from 26 to 207, with a median of 101 (IQR 79, 112). An estimated 25% of the constituents agreed to provide transportation for the cats the day after surgery. Prior to the intervention in 2017, the median percent of kittens aged <5 months was 52% of all cats entering the shelter (IQR 51, 53l). After the intervention, the percent of kittens increased (*P* = 0.001) to a median of 61% (IQR 61, 64). However, the percent of kittens entering the shelter with an outcome of euthanasia (Figure 3) decreased (*P* < 0.001), from a median of 59% (IQR 54, 73) to a median of 3% (IQR 1, 3). A median of 686 (IQR 548, 960) kittens were euthanized per year prior to intervention, and 27 (IQR 26, 28) per year after. This resulted in the median LRR for kittens increasing (*P* = 0.001) from a median of 34% (IQR 23, 38) prior to 2017 to a median of 92% (IQR 91, 92) after intervention. Death in shelter for kittens increased (*P*= 0.001) after the intervention from a mean of 2% (SD 1) to a mean of 4% (SD 1). A median of 23 (IQR 17, 30) kittens died in shelter per year prior to intervention, and 42 (IQR 41, 70) after. Death in shelter for adults was 1% (SD 0) before and 1% (SD 1) after intervention and was not different (*P* = 0.085).

### Field Services Transition

**Figure 4:**
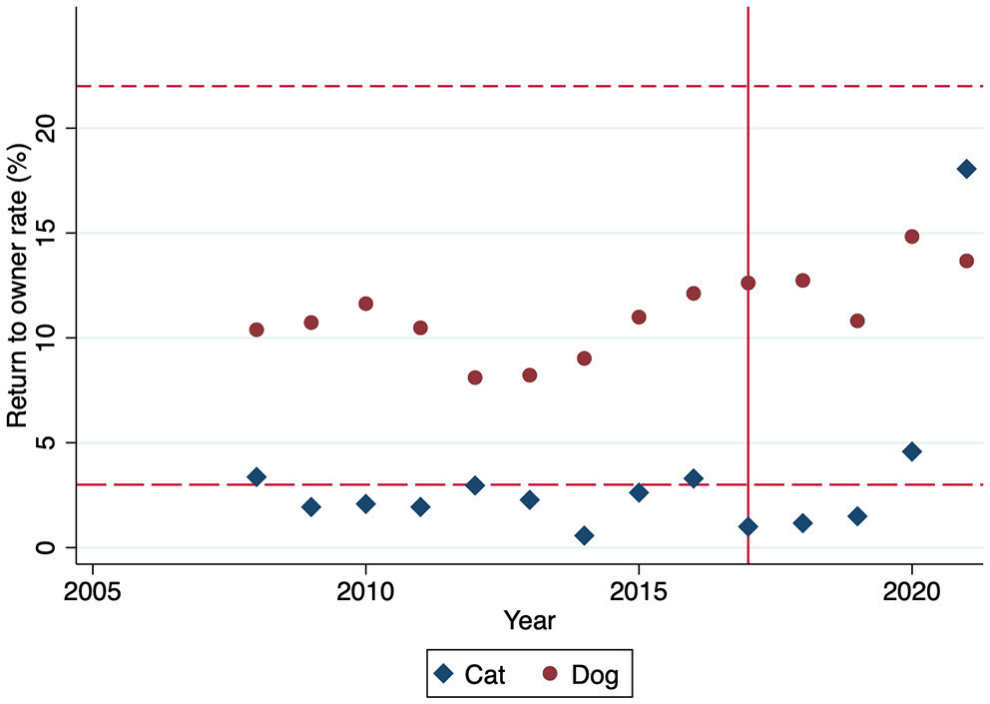
Return to owner rate by year for cats (blue diamond) and dogs (red dot). Solid line at 2017, the year the community case study began. Short dashed line at the national average dog return to owner rate (22%), and long dashed line at the national average cat return to owner rate (3%).

Between 2008 and 2016, the RTO rate for cats (Figure 4) was a median of 2% (IQR 2, 3). After the intervention, the RTO rate for cats was a median of 2% (IQR 1, 5). The RTO rate for cats was not different after the intervention (*P* = 0.898), and the median RTO rate was below the national average of 3% for cats for all but 1 year (7).

For dogs between 2008 and 2016, the median RTO rate (Figure 4) was 10% (IQR 9, 11). From 2017 to 2021, the RTO rate for dogs increased (*P* = 0.007) to 13% (IQR 13, 13). Median RTO rate for dogs was below the national average of 22% at all time points (7). There were 161 dogs reunited in the field with their owner that were not reflected in intake or RTO numbers in 2021. For comparison, in 2021 there were 518 dogs admitted to the shelter with an outcome of RTO. An internal analysis using ArcGIS Pro conducted by MAS of addresses for 328 dogs with an intake type of stray, outcome type of return to owner, and mappable found and reclaimed addresses between July 2020 and December 2021 found that the median distance from the owner's home address to the stray pick-up location was only 0.5 miles for dogs reclaimed from MAS (range 0 to 18.3 miles).

In an analysis of the 9,991 field service calls received in 2019 and 2020, 5,425 (54%) were for stray roam, 2,422 (24%) were for stray aggressive, 1,302 (13%) were for bite/dangerous, and 842 (8%) for welfare investigation.

### Streamlined Adoption and Transfer

**Figure 5:**
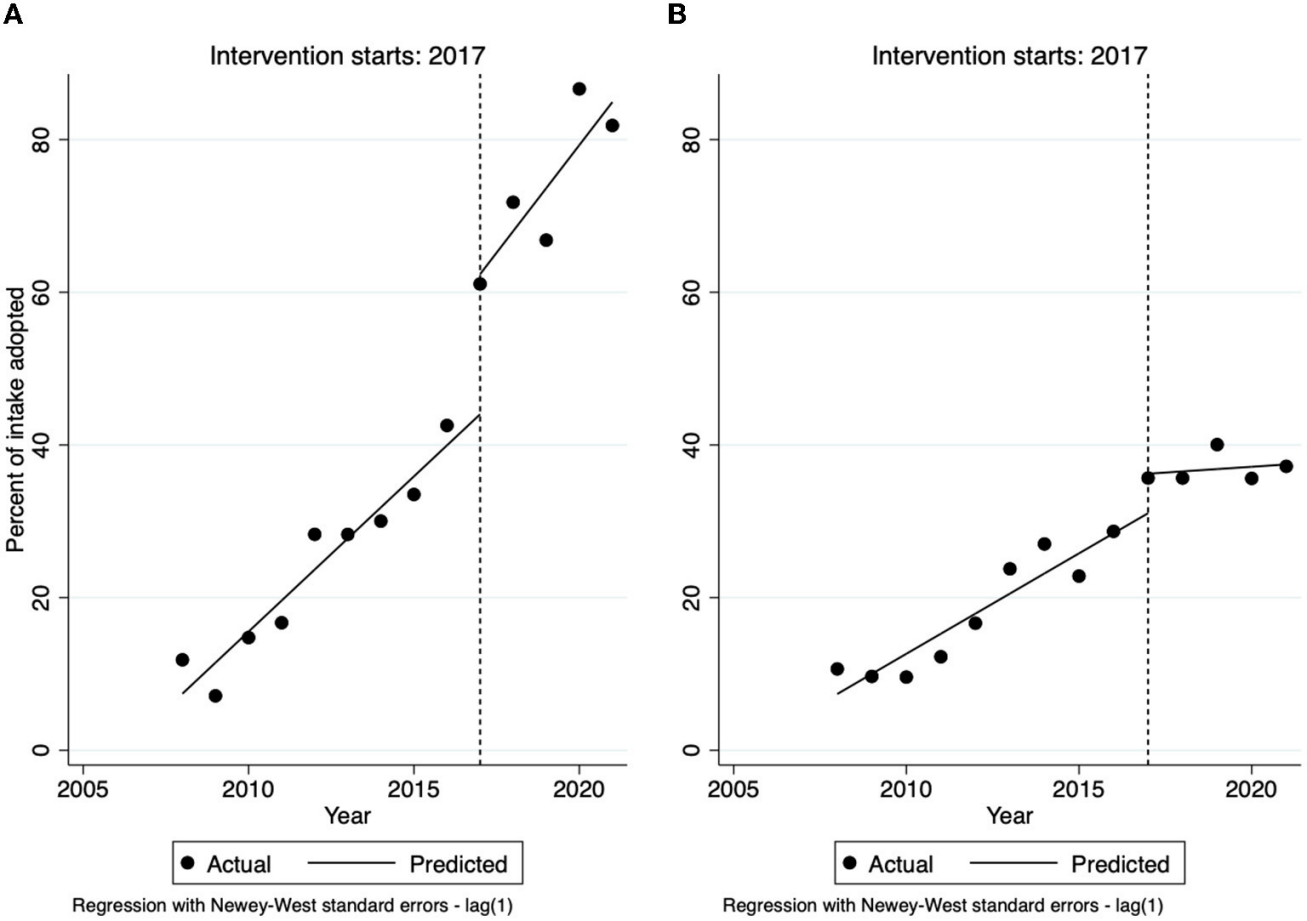
Interrupted time series analysis of the percent of intake of cats **(A)** and dogs **(B)** with an outcome of adoption. Line at intervention year (2017).

The percent of cats entering the shelter that had an outcome of adoption prior to 2017 was a median of 28% (IQR 15, 30). After the intervention, the percent with an outcome of adoption increased (*P* = 0.001) to a median of 72% (IQR 67, 73). The percent of adoption outcomes had linearly increased 4% per year prior to 2017 (*P* < 0.001), but an interrupted time-series analysis (Figure 5A) demonstrated that there was a 20% increase in the percent of adoptions immediately after the intervention (*P* < 0.001), after which adoptions continued to increase 4% per year (*P* = 0.032). The percent transferred increased from a median of 6% (IQR 5, 13) to 13% (IQR 11, 15), although this was not significant (*P* = 0.298).

The percent of dogs entering the shelter that had an outcome of adoption prior to 2017 was a median of 17% (IQR 11, 24). After the intervention, the percent with an outcome of adoption increased (*P* = 0.001) to a median of 36% (IQR 36, 36). Interrupted time series analysis of percent of dog adoption (Figure 5B) showed that adoptions were increasing at 3% per year prior to 2017 (*P* < 0.001), there was an increase of 6% immediately the intervention (*P* = 0.034), and then adoptions decreased by 3% per year after 2017 (*P* = 0.001). The percent transferred increased (*P* = 0.004) from a median of 3% (IQR 1, 15) to 39% (IQR 38, 41).

## Discussion

Implementation of best practices helped MAS to eliminate the long-standing correlation between intake and euthanasia, resulting in a live release rate of over 90% for cats and nearly 90% for dogs even though overall intake did not decrease after the intervention. The improvement in community trust may increase intake if community members are no longer fearful that healthy pets will be euthanized (24). While there were positive trends in measures such as the live release rate prior to the intervention, implementation of best practices dramatically accelerated progress toward the goal of at least 90% live release rate. The implementation of best practices was made easier by the movement of MAS from the Parks and Neighborhoods department to an independent department and reclassifying the shelter director position as a director position as this change removed several layers of decision-makers between the shelter administrator and the mayor.

### Managed Strategic Intake

MAS leadership found that the service of intaking owned pets was not required by statute or contract and determined that accepting an owner surrender, particularly when an owner just needed temporary assistance, was not in the best interest of the shelter, the pet, or the pet owner. Persons surrendering animals due to temporary hardships are likely to acquire another pet when they were able to (25) but, in the meantime, the responsibility of keeping a surrendered pet healthy and finding a live outcome falls to the shelter. While there was initial concern from animal advocates that people would abandon their pets if they were not admitted to the shelter without delay, the shelter administration determined that non-emergent immediate owner surrender was not in line with the established goals of lifesaving, public safety, and animal welfare, was not fiscally responsible and did not create sustainable resolution. No increase in abandonment was noted by the shelter administration, an experience similar to other shelters implementing managed intake (26). The change to managed intake also provided a platform for a proactive conversation about the perceived need to surrender the pet to the shelter and offered an opportunity for interventions such as the pet owner safety net.

### Pet Owner Safety Net

There was a decrease in owner surrendered dogs and cats after the implementation of the safety net program. For cats, this decrease was the only significant change in the composition of intake type. The proportion of cats younger than 5 months increased, likely due to a decrease in adult owner surrenders. For dogs, there was an increase in both stray and “other” intake. It is unknown whether this increase in stray intake for dogs was related to the decrease in owner surrender, for example if owners claimed that their dog was stray or abandoned their dog.

### Community Cat Return to Field

Despite relatively small numbers the community cat program was impactful in several ways. First, it reserved vital resources such as shelter space and human capital for cats who had no other options by providing a live outcome within a very short period at the shelter for cats that could be returned. Secondly, it returned cats to the location they were found so that lost owned cats would have a greater chance of being reunited with their family, unowned cats would have a chance to be directly adopted into a new home by the finder of a stray cat, and unsocial community cats were returned to their outdoor home. A key aspect of this program was the recruitment of finders to return the cats to their original location the day after surgery as it reduced the workload for the staff. Another important benefit for the ACOs and other staff was the reduction in the number of healthy cats they were assigned to euthanize.

The percent of kittens with an outcome of death in shelter increased from 2 to 4%, likely due to kittens <8 weeks of age, that are more likely to die in care, no longer being euthanized on intake. Recent studies of shelter mortality for kittens younger than 8 or 9 weeks have found rates of 12.6% (95% CI 10.8, 14.4) (27) and 2.5% (95% CI 0.8, 5.7) (28), respectively. The rate of 1% did not change for adult cats, supporting that this increase may be due to increased mortality for very young kittens. In absolute numbers the median number of kittens that died or were euthanized per year prior to intervention was 875 as compared to 66 after; while there is always a concern regarding animal welfare when death in shelter increases, the increase here is consistent with mortality rates observed in other shelters with programs that support young kittens and does not support the argument that over 800 kittens per year would have been better off euthanized.

### Field Services Transition

The animal services field team is a key shelter resource. Since a large percentage of calls for service do not involve public or animal safety issues, there is opportunity to deploy the PRC team to intervene and find alternatives to shelter intake. This change would conserve shelter resources and allow the field team to focus on true safety issues. Future goals for the Pet Resource Center include dedicated staff responding in person to calls for a dog at large when the finder is unable to foster, prioritizing ACO time for true public and animal safety cases and making the best use of shelter resources. This approach would parallel the evolution seen with emergency services for people in Memphis. Dispatch personnel for 911 are trained to prioritize calls for a medical team vs. a call that can be handled by a nurse practitioner, and determine which calls are not emergent and can wait for assistance.

The shift in mindset from enforcement to assistance proved to be one of the most challenging changes to implement at the shelter since ACOs had always believed they were doing what was in the best interest of the dog at large and the community. During the initial transition from a punitive, enforcement-minded field and shelter team to one of inclusivity, compassion, and providing direct assistance, staff who were not willing to adapt were transitioned to other opportunities.

The RTO rate for MAS for cats was lower than the national average for most years. The last 2 years of data showed an artifactual increase in the RTO rate that was due to the denominator of stray intake dramatically decreasing due to a change in the intake type for kittens from stray to wildlife. The RTO rate for dogs was significantly lower than the national average for all years and there were fluctuations both before and after the intervention. The RTO rate after the intervention may have been artifactually lower due to the return of stray dogs in the field by ACOs prior to shelter intake and possibly the effect of dogs that were misclassified as stray by an owner wishing to surrender.

### Streamlined Adoption and Transfer

The percent of adoption outcomes increased for both cats and dogs. For cats, this increase in adoption outcomes was likely due to the elimination of euthanasia of underage kittens, most of which had an outcome of adoption after the intervention. For dogs, adoptions initially increased after the intervention, which may have been due to the elimination of requirements such as a home check for Pitbull type dogs and a streamlined adoption process. The slight decrease in adoption over time after the intervention may be due to competition from transfer, which increased dramatically. The increase in transfer may be due to the elimination of the transfer out fee.

## Limitations

Consistent with national trends (7), there was a large decrease in intake and changes to shelter operations during 2020 that complicated trend analysis. Only 5 years of data were available after the intervention, complicating statistical analysis through bias toward the null. There was a transition in shelter software in 2020 that changed how some animal types, particularly the intake type of neonatal kittens, were classified. It was not possible to determine whether animals classified as stray were actually owner surrender or abandoned. Multiple programs were implemented and refined over different time periods, making it impossible to quantify the impact of individual interventions. Secular trends such as increases in live release were already present and may not have been fully statistically controlled. However, despite these limitations, the dramatic decoupling of euthanasia from intake demonstrates that there was truly a difference after intervention beyond the continuation of secular trends.

Future research should attempt to look at longer periods of time (at least 7 years) and would ideally control the implementation of programs so that the individual impact of different programs can be determined. More data is required to determine the impact of pet owner safety nets on pet retention. There should also be an effort to determine whether making owner surrender less convenient, whether through scheduled appointments or other interventions that owners perceive as barriers, results in an increase in stray intake at other locations and if so whether some portion of the stray animals are truly owner surrender or abandoned.

## Conclusion

Implementation of these best practices accelerated MAS' progress toward a live release rate over 90%, dramatically decreased kitten euthanasia, increased the RTO rate for dogs and severed the historical correlation between euthanasia and intake.

## Data Availability Statement

Publicly available datasets were analyzed in this study. This data can be found here: https://www.memphistn.gov/animal-services/shelter-statistics/.

## Author Contributions

SP: conceptualization, writing—original draft, supervision, and project administration. AP: resources and writing—original draft. RK: formal analysis, data curation, writing—original draft, and visualization. All authors contributed to the article and approved the submitted version.

## Conflict of Interest

SP was the lead consultant for Target Zero and completed the pro bono 2016 assessment for Memphis Animal Services. SP is the author of the Best Practice Playbook for Animal Shelters book referenced in this manuscript. Her company Team Shelter USA is sponsoring the page charges for this submission. AP and KP were both employed by Memphis Animal Services. The remaining author declares that the research was conducted in the absence of any commercial or financial relationships that could be construed as a potential conflict of interest.

## Publisher's Note

All claims expressed in this article are solely those of the authors and do not necessarily represent those of their affiliated organizations, or those of the publisher, the editors and the reviewers. Any product that may be evaluated in this article, or claim that may be made by its manufacturer, is not guaranteed or endorsed by the publisher.

## Footnotes

^1^ShelterLuv, ShelterLuv, Inc., Palo Alto, CA.

## References

[1] Zawistowski SMJ. The evolving animal shelter. In: Shelter Medicine for Veterinarians and Staff . 1st ed. England: Oxford (2004). p. 3–9.

[2] The Humane Society of the United States. Pets by the Numbers: US. Pet Ownership, Community Cat and Shelter Population Estimates. (2020). Available online at: https://humanepro.org/page/pets-by-the-numbers (accessed September 9, 2021).

[3] Velasco-Villa A Reeder SA Orciari LA Yager PA Franka R Blanton JD. Enzootic rabies elimination from dogs and reemergence in wild terrestrial carnivores, United States. Emerg Infect Dis. (2008) 14:1849–54. 10.3201/eid1412.080876 19046506 PMC2634643

[4] Velasco-Villa A Escobar LE Sanchez A Shi M Streicker DG Gallardo-Romero NF. Successful strategies implemented towards the elimination of canine rabies in the Western Hemisphere. Antivir Res. (2017) 143:1–12. 10.1016/j.antiviral.2017.03.023 28385500 PMC5543804

[5] NACC. National Animal Care and Control Association Guidelines. (2019). Available online at: https://www.nacanet.org/wp-content/uploads/2019/03/NACA_Guidelines.pdf (accessed February 20, 2022).

[6] Best Friends Animal Society. Humane Animal Control Effective Enforcement, Shelter Management, Local Governmane Support and Community Engagement. Kanab, UT: Best Friends Animal Society (2019).

[7] Best Friends. The State of U.S. Animal Sheltering. (2020). Available online at: https://network.bestfriends.org/research-data/research/state-us-animal-sheltering-2020 (accessed December 30, 2021).

[8] Pizano S. Best Practices Playbook for Animal Shelters. (2021). 1st ed. Coral Springs, FL: Team Shelter USA.

[9] Lord LK Wittum TE Ferketich AK Funk JA Rajala-Schultz PJ. Search and identification methods that owners use to find a lost cat. J Am Vet Med Assoc. (2007) 230:217–20. 10.2460/javma.230.2.217 17223754

[10] Lord LK Wittum TE Ferketich AK Funk JA Rajala-Schultz PJ. Search and identification methods that owners use to find a lost dog. J Am Vet Med Assoc. (2007) 230:211–6. 10.2460/javma.230.2.211 17223753

[11] ASPCA. Pet Statistics. (2021). Available online at: https://www.aspca.org/helping-people-pets/shelter-intake-and-surrender/pet-statistics (accessed 30, 2021).

[12] Kremer TA. New web-based tool for RTO-focused animal shelter data analysis. Front Vet Sci. (2021) 8:669428. 10.3389/fvets.2021.669428 34113674 PMC8185155

[13] Marsh P. Replacing Myth with Math: Using Evidence-Based Programs to Eradicate Shelter Overpopulation. (2010). Concord, NH: Town and Country Reprographics, Inc. p. 8.

[14] Zito S Vankan D Bennett P Paterson M Phillips CJC. Cat ownership perception and caretaking explored in an internet survey of people associated with cats. PLoS ONE. (2015) 10:1–21. 10.1371/journal.pone.0133293 26218243 PMC4517794

[15] Best Friends. Managed Intake or Admissions Training Playbook. (2021). Available online at: https://network.bestfriends.org/education/manuals-handbooks-playbooks/managed-intake-or-admissions-training-playbook (accessed December 30, 2021).

[16] National Animal Care Control Association. NACA Guideline on Appointment-Based Pet Intake into Shelters. (2021). Available online at: https://www.nacanet.org/author/me_fa9mql12/ (accessed December 30, 2021).

[17] Weiss E. Safety nets and support for pets at risk fo entering the sheltering system. In: Animal Behavior for Shelter Veterinarians and Staff . 1st ed. New York: Wiley (2015). p. 286–91.

[18] Spehar DD Wolf PJ. The impact of an integrated program of return-to-field and targeted trap-neuter-return on feline intake and euthanasia at a municipal animal shelter. Animals. (2018) 8:55. 10.3390/ani8040055 29652808 PMC5946139

[19] Edinboro CH Watson HN Fairbrother A. Association between a shelter-neuter-return program and cat health at a large municipal animal shelter. JAVMA. (2016) 248:298–308. 10.2460/javma.248.3.298 26799109

[20] Holland KE. Acquiring a pet dog: a review of factors affecting the decision-making of prospective dog owners. Animals. (2019) 9:124. 10.3390/ani9040124 30925784 PMC6523466

[21] Veazey K. First Word: Animal Shelter as an Issue. Commercial Appeal. (2021). Available online at: https://archive.commercialappeal.com/columnists/kyle-veazey/first-word-animal-shelter-as-an-issue-more-strickland-nuggets-mongo-returns-ep-1181940052-324354971.html (accessed December 30, 2021)

[22] Memphis Animal Shelter. Shelter Statistics—City of Memphis. (2020). Available online at: https://www.memphistn.gov/animal-services/shelter-statistics/ (accessed December 30, 2021).

[23] Scarlett JM Greenberg M Hoshizaki T. Every Nose Counts Using Merics in Animal Shelters. I Maddie's Fund. Scotts Valley, CA: CreateSpace Independent Publishing Platform (2017).

[24] Spehar DD Wolf PJ. The impact of return-to-field and targeted trap-neuter-return on feline intake and euthanasia at a municipal animal shelter in Jefferson county, Kentucky. Animals. (2020) 10:1–18. 10.3390/ani10081395 32796681 PMC7459743

[25] Scarlett JM Salman MD New JG Jr Kass PH. Reasons for relinquishment of companion animals in US animal shelters: selected health and personal issues. J Appl Anim Welf Sci. (1999) 2:41–57. 10.1207/s15327604jaws0201_4 16363961

[26] Carr B. Cats by Appointment Only. (2021). Available online at: https://www.maddiesfund.org/cats-by-appointment-only.htm (accessed Febraury 20, 2022).

[27] Dolan ED Doyle E Tran HR Slater MR. Pre-mortem risk factors for mortality in kittens less than 8 weeks old at a dedicated kitten nursery. J Feline Med Surg. (2020) 23:730–7. 10.1177/1098612X20974960 33252306 PMC10812196

[28] Berliner EA Scarlett JM Cowan AC Mohammed H. A prospective study of growth rate, disease incidence, and mortality in kittens less than 9 weeks of age in shelter and foster care. J Appl Anim Welf Sci. (2022) 2021:1–16. 10.1080/10888705.2021.2021409 34994268

