# Supplementary material for: The Impact of Incorporating Multiple Best Practices on Live Outcomes for a Municipal Animal Shelter in Memphis, TN
Source: Front Vet Sci. 2022 Jun 24;9:786866. doi: 10.3389/fvets.2022.786866 (PMC9263921; doi:10.3389/fvets.2022.786866)
Supplement: Supplementary file 1 [file Data_Sheet_1.pdf]

TIPS FOR CONTACTING RESCUE GROUPS

- Be patient and polite! Most rescue groups are run 100% by volunteers with full-time jobs.
- Contact them ONLY if your pet fits their intake criteria.
- Send as much information about your pet as possible, including:
  - Multiple photos
  - Age, Weight, Sex, Breed
  - Spayed or Neutered?
  - Vaccination & Heartworm Status
  - Temperament: Does your pets get along with adults? Kids? Dogs? Cats? Share anything you know about your pet's personality.
- Offer to foster if at all possible! Most groups don·t have a facility, so they rely on foster homes to be able to take in a pet. If you can keep your pet while they try to find a new home for him, or even just for a few days while they look for a foster, that can bea hugehelpl
- If you·re willing to help pay for costs associated with rehoming your pet, like medical bills, food. or supplies, let them know that! Most rescues run completely on donations.

NO LUCK WITH LOCAL RESCUE GROUPS?

Go onlineto Rehome.AdoptAPet.com for a safe, no-cost way to rehome your pet.

See below for more info from their website.

How It Works

We believe that rehoming pets should be easy and stress free both for you and your pet Our expeJts at Adopt-a-Pet com , the larg est non-prnfit pet adoption websi te , with support from The Petco Foundation, have created a simple, r iabl e, frt"ie p!'ogram 1..-. help you place your pet fr0tn yOJJr lovir.g home directly to another

1 **Create A Pet Profile**
Get your pet posted on our site to be seen by mill1oos of visit ors each month.

2 **Review Applications**
Don't wooyl Our dedicated team will give you all the help you need to select the best pol!!fltial adop!era

3 **Meet Adopters**
We'll guide you through the process of setting safe and pressure-free meetings with applicants

4 **Finalize Adoption**
We'll provide you with an adoption contract to protect lhe transfer of your pet ownership.

SKIP THE SHELTER!

Help ensure a happy ending for your pet by worh.ing with a rescue group!

MEMPHIS ANIMAL SERVICES

2350 APPLING CITY CV., MEMPHIS, TN 38133
901-636-1416 | MEMPHISANIMALSERVICES.COM

In review

DOGS ONLY

BREED-SPECIFIC DOG RESCUES

Belly Rubs Basset Rescue
Email: bellyrubsbr@b ellyrubs.org
Basset hounds & basset hound mixes

Boston Terrier Rescue of East TN
Phone: 865-983-3272
Email: rjraible y@yahoo.com
Boston Terriers, Pugs, & French Bulldogs

Bullmastiff Rescuers, Inc.
Email: leia.peia1@y ahoo.com
Buimastiffs

Imminent Danger German Shepherd Rescue
Online owner surrender form:
http://members.petfinder.com/ ~TN668/0 TI.html
Email: imindangergs rescue@gmail.com
German Shepherds

Memphis Area Golden Retriever Rescue
Email: intake @magrr.org
Golden Retriever or Golden Retriever mixes. Must be friendly to people & dogs.

Mid-South Pug Rescue
Phone: 901-209-9784 / 901-488-9195
Purebred pugs

North Mississippi Great Dane Rescue
Phone: 901-461-5997
Email: nmsgdr @hotmail.c om
Great Danes or Great Dane mixes

Tails of Hope Dog Rescue
tailsofhopedr @yahoo.com
English Bulldogs, German Shepherds, Pomeranians, Dachshunds, Yorkies , French Bulldogs, Pugs, Puggles, Boston Terriers (You may also contact them about pigs, chickens, & goats!)

West Tennessee Border Collie Rescue

DOGS ONLY

GENERAL DOG RESCUES

A Little Bit of Snuggles/HappiDog Rescue
Email: happidogrescue@gmail.com
Send an email to let them know if the dog is people-friendly, dog-friendly, up-to-date on vaccines, heartworm-positive or heartworm-negative, age, current housing status (indoor or outdoor). Photos are also helpful.

Real Good Dog Rescue
Phone: 865-652-9781
Email: ltrenthem@gmail.com
If you're able to keep your dog for a few days, Real Good Dog Rescue may be able to take them. They often need a few days to find a foster or adopter.

Wilson Animal Rescue
Email: info@wilsonanimalrescue.com
Dog- and kid-friendly required. Wilson is open to any breed , age, and medical status. They will need time to find a foster, and you can help by offering to hold on to your dog for a few days to a week.

Guardian Angel Pet Rescue
Email: debbie.homas @gmail.com
Phone: 901-568-9327
Smaller dogs

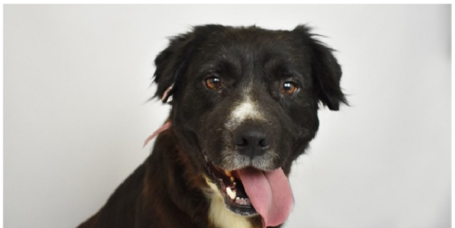

BOTH DOGS & CATS

GENERAL DOG & CAT RESCUES

Meadow Wood Rescue
Email: syoung0500@a ol.com
Specializes in elderly or injured dogs & cats

Perfect Match Animal Rescue
Email: pmar.application@gmail.com
Can often advise and assist with programs to help, training methods, supplies & pet expenses. PMAR tries to take as many pets as space and foster homes allow.

Sunny Meadows
Email: tnsafehavenforpets @yahoo.com
Sunny Meadows will try to help when they have space. Smaller dogs are more likely to be accepted due to space.

Wright-Way Rescue
Email: cou rtney.stone@wright-wayrescue.org
Takes all breeds of dogs , cats, puppies and kittens (and moms) on space-available basis. Priority given to pets with good temperaments.

CATS ONLY

GENERAL CAT RESCUES

Kitty City/LUCRA
Email: leggup@g mail.com
They are not able to take your cat from you, but they welcome owners needing to rehome their cat to bring fully tested , spayed/neutered, and vaccinated cats to their adoption events at Hollywood Feed on Saturdays and Sundays to help find them a new home NOTE: Registration is required due to space. Email the address above to register.

Mewtopia
Phone: 901-503-9328
May be able to help place younger cats [illegible]
