# Supplementary material for: The Impact of Incorporating Multiple Best Practices on Live Outcomes for a Municipal Animal Shelter in Memphis, TN
Source: Front Vet Sci. 2022 Jun 24;9:786866. doi: 10.3389/fvets.2022.786866 (PMC9263921; doi:10.3389/fvets.2022.786866)
Supplement: Supplementary file 2 [file Data_Sheet_2.pdf]

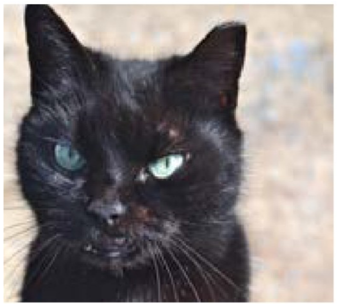

SOLVING YOUR CAT ISSUES

Cats are sleeping somewhere on m!:J propert!:j

They're looking for somewhere safe and dry to take shelter. You can either provide a shelter, like a dog house, hidden away, or you can block open areas with lattice or chicken wire (make sure no animals are inside before doing this).

I smell cat urine

This is most likely a male cat (tomcat) "spraying ," which is reduced once a cat is neutered, and the odor of their urine is also less pungent.

Feeding cats attracts pests

This won't be an issue if cat food is only put out at designated times during daylight hours and picked up before twilight. Cat food messes should be picked up to avoid pests feeding on it. Also, cats hunt and actually help with pests!

Cats are loud, fighting, and having ltittens

Spaying/neutering will nip these breeding behaviors in the bud. TNR these cats if they 're not owned! Spay/neuter will reduce the hormones causing these nuisance behaviors.

TNR WITH MEMPHIS ANIMAL SERVICES

Trap - Neuter - Return

Memphis Animal Services offers FREE community cat spay/neuter surgery to all Memphis city residents (plus Snelby County residents excluding Bartlett, Germantown, & Collierville).

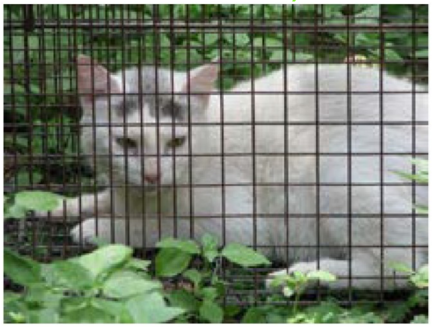

Community Cat Intake Hours

Tuesday through Saturday from 12-4 p.m.
You must be in line by 3:30 p.m.

Let us know if you'd like to volunteer to return the cat to where you found him after he's recovered from spay/neuter surgery.

COMMUNITY CAT DIVERSION

A new approach to living with community cats

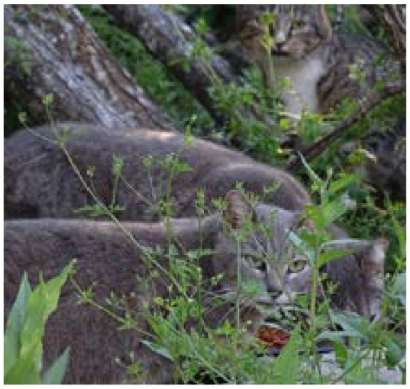

MEMPHIS ANIMAL SERVICES

2350 APPLING CITY CV.. MEMPHIS, TN 38133
901-636-1416 | MEMPHISANIMALSERVICES.COM

What is a community cat?

A community cat is any cat who lives outdoors and who does not have a particular home or owner. Some community cats are friendly and socialized, and some are not. Community cats usually depend on humans nearby for a food source, like a caretaker regularly putting out food for them, dumpster, or other source.

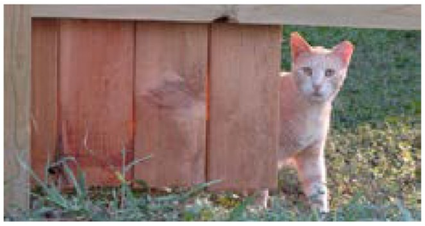

What is community cat diversion?

In community cat diversion, we acknowledge that by law, cats have the right to roam, unlike dogs, who must always be on leash or contained. We now use the progressive, humane method of "Trap-Neuter-Return" (or TNR) to control our community 's cat population. TNR is the most effective way to control community cat population growth. Cats who've been spayed /neutered and returned have a tipped left ear, as in the photo above.

r **When you become aware of a stray or loose cat (with no owner you're aware of) near your home or office, simply bring him** to us at MAS during our stray intake hours (Tuesday-Saturday 12-3:30pm). After the 3-day stray holding period, we will spay or neuter the cat and return him to where you

Why can't we just stick to the old way?

Before, MAS would simply take in every cat found outdoors who was brought to us, despite the discouraging fact that **only about 2% of cats taken to shelters are reunited with their owners.** Plus, most open-intake city shelters in our area do have to euthanize for space, so cats taken to a shelter might not even be adopted.

Also, we know now that "trap and remove" simply does not work. After decades of taking "stray" cats **to** shelters, we still don't have fewer cats living outside. This is due to the "Vacuum Effect:" if you remove cats from an area, more cats will move right in! You need to leave the existing (but now fixed) cats there to stabilize the population by protecting their territory from unfixed cats.

How does TNR help ME?

TNR is the most effective way for controlling outdoor cat populations--whether you love community cats or consider them a nuisance.

Here are some ways that TNR reduces community cat-related nuisances that "trap and remove" does NOT:

- It immediately stabilizes the size of the colony by eliminating new litters
- It greatly reduces nuisance behaviors often associated with community cats, like mating-related yowling and fighting, as well as the odor of unneutered males spraying to mark their territory.
- The spayed and neutered cat colony (or group of cats living in a certain territory) will protect that territory from other new unsterilized cats moving into it and starting the cycle all over again
- It reduces the number of cats that come into

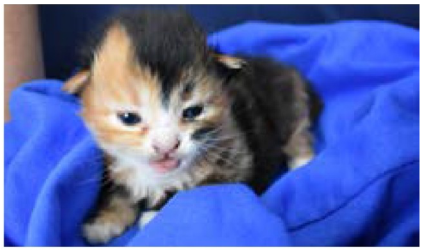

What if I find kittens?

DON'T KIT-NAP THEM!

Mom is most likely just off hunting and will return. The best way to help the kittens is to wait 2-3 hours and watch from a distance to see if Mom comes back. Kittens need their mom--don't take them from where they are unless you're certain Mom isn't coming back.

If she comes back, she can care for them until they're 8 weeks old. At that point, you can either foster and spay/ neuter Mom and kittens to find new homes, or you can bring them to MAS. We will spay Mom and return her, and we'll adopt out the kittens .

If Mom has not come back after 2-3 hours, these kittens may need your help! Bring them to **MAS** during our intake hours (Tuesday-Saturday 12-3:30pm). In the meantime, they're hungry! Go to kittenlady. org/savekittens to learn how to temporarily
