# Supplementary material for: The Impact of Incorporating Multiple Best Practices on Live Outcomes for a Municipal Animal Shelter in Memphis, TN
Source: Front Vet Sci. 2022 Jun 24;9:786866. doi: 10.3389/fvets.2022.786866 (PMC9263921; doi:10.3389/fvets.2022.786866)
Supplement: Supplementary file 3 [file Data_Sheet_3.pdf]

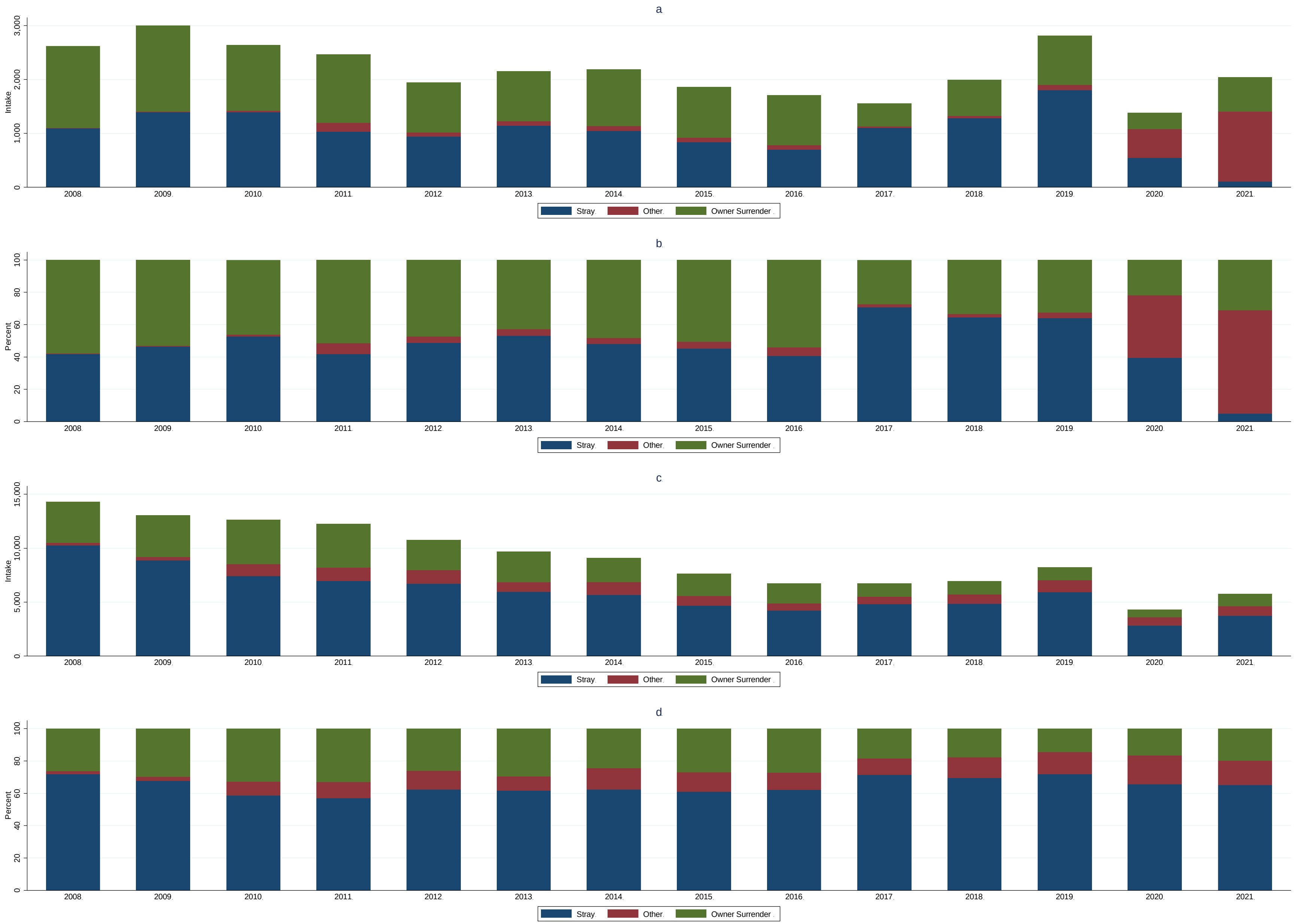
a
Intake
0
1,000
2,000
3,000
2008
2009
2010
2011
2012
2013
2014
2015
2016
2017
2018
2019
2020
2021
Stray
Other
Owner Surrender
b
Percent
0
20
40
60
80
100
c
Intake
0
5,000
10,000
15,000
d
Percent

Addendum 3 Stacked bar chart showing intake and intake type by year for cats by gross numbers (a) and as a percent of intake (b). Stacked bar chart showing intake and intake type by year for dogs by gross numbers (c) and as a percent of intake (d).
